# Barriers and facilitators to primary healthcare encounters as reported by autistic adults: a qualitative study

**DOI:** 10.3389/fmed.2025.1481953

**Published:** 2025-03-12

**Authors:** Leah I. Stein Duker, Willa Giffin, Elinor E. Taylor, Lily Shkhyan, Amber Pomponio Davidson, Laura Mosqueda

**Affiliations:** ^1^Chan Division of Occupational Science and Occupational Therapy, University of Southern California, Los Angeles, CA, United States; ^2^Department of Health and Rehabilitation Sciences, College of Public Health, Temple University, Philadelphia, PA, United States; ^3^Department of Family Medicine, Keck School of Medicine of University of Southern California, Los Angeles, CA, United States; ^4^Leonard Davis School of Gerontology, University of Southern California, Los Angeles, CA, United States

**Keywords:** autism, primary care, autistic adults, environment, qualitative

## Abstract

**Background:**

Primary care is designed to co-manage health concerns, contribute to preventive care, and provide medical care coordination. Receiving primary care may be especially vital for autistic people, many of whom disproportionately experience psychiatric and physical health conditions. However, autistic adults often face barriers to receiving primary care, and first-hand accounts of these challenges are limited. Therefore, the purpose of this study was to describe barriers and facilitators to primary care encounters as reported by autistic adults.

**Methods:**

Interviews were conducted with 34 autistic adults in Los Angeles and Philadelphia, lasted an average of 26 min, were transcribed verbatim, and analyzed using thematic analysis. This dataset is part of a larger study that interviewed autistic adults, caregivers, and primary care providers (PCPs).

**Results:**

Participants were primarily White, non-Hispanic, and had a mean age of 32 years. Five overarching themes describing challenges and potential strategies to improve care emerged from the interviews: (1) *finding a primary care provider*, (2) *the physical environment*, (3) *communication,* (4) *autism-specific knowledge,* and (5) *support for primary care encounters*, while simultaneously highlighting the importance of tailoring care for autistic adults to improve primary care experiences. Results, specifically in the communication and autism-specific knowledge themes, were consistent with the neurodiversity model of autism, as participants highlighted stigma and mutual communication as key healthcare barriers.

**Conclusion:**

Findings provide a nuanced understanding of autistic adult participants’ primary care experiences, highlighting their perception of barriers and facilitators to these healthcare encounters. These results offer valuable insights for improving the accessibility and quality of care for autistic people, many of which are practical, low/no cost, and easy to implement. Strategies also emphasized the diversity of experiences and preferences for autistic patients, highlighting the importance of tailoring accommodations in the primary care setting.

## 1 Introduction

Primary care services are designed to be patient-centered medical homes that co-manage immediate health concerns, contribute to preventive health care, and coordinate care with other providers (1). The importance of this care may be especially vital for individuals who experience elevated health risks and co-occurring conditions. Autistic people^1^ comprise one such at-risk group. Autistic individuals exhibit great heterogeneity in their clinical presentations, abilities, support needs, and degrees of functional impairment (2). However, core autistic characteristics include: differences or challenges with social communication; strong preferences for routines; repetitive motor movements; sensory sensitivities; and focused interests of intense, persistent fixation (2).^2^ When compared to the general population, autistic people disproportionately experience psychiatric and physical health conditions (3–6), as well as premature mortality by 16–30 years (7).

Although our understanding of the healthcare experiences of autistic people across the lifespan has grown, past research has predominantly focused on the needs of children (8, 9), with reports of numerous obstacles to the receipt of primary care for autistic children and their caregivers (e.g., 10–12). However, autistic adults also commonly face barriers to receiving primary care at the patient-, provider-, and system-levels (e.g., 13). These include, but are not limited to: sensitivity to the physical environment; communication difficulties; anxiety due to waiting, sensory over-stimulation, and mutual communication challenges between patients and providers; autism-related stigma; lack of provider autism-specific knowledge; and problems with accessibility and the complexity of the healthcare system (e.g., 14, 15). Much of the current research centers non-autistic caregiver or provider voices (e.g., 16–22), with burgeoning research from the perspective of autistic adults (e.g., 23–25), or multiple informant groups (e.g., 13, 26). As the population of autistic adults continues to grow (27), a first-hand accounting of the factors hindering and promoting successful primary care encounters for autistic adults is essential. Therefore, the purpose of this study was to describe the barriers and facilitators to primary care as reported by autistic adults.

## 2 Methods

### 2.1 Design

This study was part of a larger mixed-methods study to explore barriers and facilitators to primary care for autistic adults from the perspective of autistic adults, caregivers of autistic adults, and primary care providers (PCPs). Reported here are the qualitative results from interviews with autistic adults; results from caregivers were reported in another publication (18). Ethics approval was received from the University of Southern California (HS-17-00477); written consent was obtained from participants at the time of data collection.

### 2.2 Participants and recruitment

Individuals 18+ years with at least one prior primary care visit were eligible to participate if they had a self-reported autism diagnosis [confirmed by medical documentation or Ritvo Autism Asperger Diagnostic Scale-Revised score (28)] and were able to communicate in English or Spanish, using verbal, written, or augmentative and alternative techniques.

Brochures/flyers were posted and presentations given at multiple sites in the Southern California and Philadelphia areas, including support groups, occupational therapy locations, and supported employment programs. In addition, two autistic collaborators posted on social media and invited autistic adults known to them. Individuals interested and eligible were enrolled consecutively.

### 2.3 Data collection

Following best practices in autism research, which recommend including autistic perspectives throughout every stage of the research processes (29), two autistic co-researchers helped develop semi-structured interview guides. Additionally, one expert in adult primary care and another in qualitative methodology also supported script development. Five autistic adults pilot tested open-ended narrative questions, with subsequent revisions made to improve clarity. The final guide included 10 questions, which inquired about primary care experiences and accommodation recommendations. Probes were used when appropriate to elicit additional information. Each interview was digitally recorded and professionally transcribed verbatim; field notes composed during interviews supplemented transcripts.

The interview team consisted of three team members with didactic and *in vivo* interview training, supplemented by study-specific training. All interviews were one-on-one, with the exception of two group interviews of participants already known to each other who requested to be interviewed together. Interviews were conducted at locations convenient to the participants (e.g., home, library); each participant was provided $30.

### 2.4 Data analysis

Inductive thematic analysis (30) was used to analyze and interpret interviews to describe autistic adults’ primary care experiences. First, two team members independently read and coded three transcripts to develop a provisional list of codes. After discussion and edits to the provisional codebook, three more transcripts were independently read and coded to identify additional codes. Following consensus, the codebook was formalized and all interviews were double-coded using QSR International’s NVivo software; discussion with a third team member occurred to resolve any discrepancies in coding. To bolster the trustworthiness of the results, multiple strategies were employed, including consensus-driven thematic development; analytic triangulation (independent co-coding); negative case analysis; establishing an audit trail, including memo writing; and fidelity checks for interview content and technique (31). Authors utilized the Standards for Reporting Qualitative Research [SRQR; (32)] for reporting; see Supplementary Table 1.

### 2.5 Positionality

The research team consisted of 10 team members, providing a diversity of perspectives and expertise. Three members identified as autistic; all others identified as non-autistic at the time the research was conducted. Seven members have experience working with autistic people, five are occupational therapists with clinical and research experience, one is a practicing PCP, and two have a background in public health.

## 3 Results

Thirty-four autistic adults enrolled in the study. Two participants were excluded when they verbally requested to end the interview after the first question (i.e., “I’m grumpy…No, mom, it’s over.”); to respect their preferences interviews were immediately concluded. Included participants were a mean age of 32 (±12) years and primarily identified as male, White, not Hispanic/Latino, spoke English in the home, and had completed college; see Table 1. Demographic information stratified by recruitment location (Los Angeles vs. Philadelphia) is provided in Supplementary Table 2.

**Table 1:** Descriptive characteristics of participants.

		Autistic participants (*n* = 32)
		Mean (SD) / Range
Age		31.8 (12.2) / 18–67 years
RAADS-R Score^a^		119.1 (51.0) / 65–224
All sources considered, how much money do you have access to monthly?^b^		$1,515.00 (1,261.00) / $0–$5,000
		***N* (%)**
Sex		
	Male	26 (81.3)
	Female	6 (18.8)
Race^c^		
	White, Caucasian	23 (71.9)
	Black or African American	4 (12.5)
	Asian	4 (12.5)
	American Indian or Alaska Native	3 (9.4)
	Not Reported	1 (3.1)
Ethnicity		
	Not Hispanic, not Latino	26 (81.3)
	Hispanic, Latino	6 (18.8)
Primary language spoken in the home		
	English	28 (87.5)
	Spanish	2 (6.3)
	More than one primary language^d^	2 (6.3)
Highest level of education earned		
	High School or GED	11 (34.4)
	College	18 (56.3)
	Graduate Degree or above	3 (9.4)

RAADS-R, Ritvo Autism Asperger diagnostic Scale-Revised.

a Autism diagnosis confirmed either by medical documentation or RAADS-R; *n* = 23 RAADS-R scores included here.

b Missing data (*n* = 9).

c Participants instructed to mark all that apply.

d English and Spanish (*n* = 1); English and Cantonese (*n* = 1).

Over 75% of participants reported good-excellent overall health, had a current primary care provider, and brought a support person to appointments. Four participants requested their caregiver be interviewed, so a small number of dyads are present in the overall data (18).

Overall, five themes emerged: (1) finding a primary care provider, (2) physical environment, (3) communication, (4) autism-specific knowledge, and (5) support. See Table 2 for additional quotes.

**Table 2:** Additional and full-context supporting quotes.

Theme	Sub-theme	Example quotes
Finding a primary care provider	Finding a PCP	“It’s like, my barber just passed away. I mean, he’s the only one that’s touched my hair for 30-some years, you know? …Same way with a doctor.”
	Objective characteristics	“It’s not the doctors, it’s the insurance that has been [causing] a lot of trouble lately…insurances have been changing a lot of regulations in the recent years. It’s been making it very difficult.”
		“Without my ability to drive…transportation would’ve been a problem.”
		“…it’s a little far [to get to the doctor’s office]…because we go by walking.”
		“…a lot of doctors do not understand that it gets cold out there and you cannot go out there…you cannot get on a bus.”
	Subjective characteristics	“He got good reviews, so I just picked him.”
		“Finding one who’s competent and knows how to read me properly.”
	Strategies	“…recommendations from others [non-family members] tends to be how I find the doctors I end up with.”
		“I had to, basically, interview a lot of them…I had to do it myself.”
		“[My PCP] helped me finally find a really good one who worked with kids on the spectrum before she became a gynecologist, so she knows sensory really, really well and is super gentle and really good with me.”
Physical environment	Sensory sensitivities	“[The paper gown] envelopes you. It’s not soft. It’s like it’s got a texture to it that’s really rough…I do not like the feeling of it, do not like the sound of it.”
		“They always have the brightest spotlights on your face.”
		“The lights are fluorescent…they give me a headache.”
		“I can feel the harshness of the fluorescent bulbs.”
		“When I’d get in the room, it was really overwhelming, all the lights and everything. And so I’d always turn them off…[My previous PCP] would always say, ‘Oh, it’s dark in here. You want those off?’…[But] the new guy, he just turns them all on and does not even ask me if that was what I wanted. When I try to tell him, he just kind of like ignores it…It just feels careless and like it just does not really matter. Like there’s no effort to make things easier or to make it doable.”
		Fluorescent lights “bother me when they emit a sound…any humming.”
		“Doing things like temperature or touching without saying you are going to do that results in more anxiety for me and I flinch or I jump.”
	Sensory sensitivities: strategies	“I do not feel comfortable [asking for accommodations] because I know he will not change.”
		“In the waiting room, I think they need like…dimmer lights.”
		“…like they could have like a blue light in there because I know blue light usually helps you relax and calm down a bit.”
		“I think that it would be better to have like a white noise or a calming type nature music in there so that you were not hearing everything they were telling everybody else and then you would not be worried about everybody else hearing what you were telling the doctor… But it should not be very loud because loud would bother people on the spectrum, including me; if it was too loud, it would overstimulate my sensory system and that would do no good.”
		“Definitely make [being in the physical office] a sensory sensitive experience.”
		I would like “…a waiting room that took into account sensory triggers like lighting and noise and air conditioning and, you know, things like that.”
	Waiting	“Negative experiences I’ve had are particularly the waiting time, both on the phone and in the waiting room, in general, and then, of course, waiting to be seen.”
		“…the waiting room is, of course, it’s boring, but as long as you are entertained with the magazines you are good. But waiting for the – once you get into the room and waiting for a doctor it’s like torture. It’s because you are like ‘When is he coming?’…a good 10 to 20 or ish.”
	Waiting: Strategies	“I try to make my appointment as best as possible, *even if it means delaying it*…[in order to have] the shortest wait time in the office.”
		“I always go either first thing in the morning or last thing in the afternoon.”
		“They let me tell the front desk if I’ve been waiting more than 15 min, and then his nurse will sometimes get me and put me in a room…she always tells me how many people are in front of me, but she’ll sometimes put me in a room if I’m getting too stressed…And sometimes, like, if I need labs or x-rays or something…she’ll have them come get me during that wait time so that I do not have to spend even longer there.”
		“I’m not a huge fan of TV, but that’d be nice, like something just to distract you while you wait, anything.”
		“I guess it’s like, for me, it’s like, using the cell phone to kill time.”
		“I always having an escape plan or somewhere where I can step away for a second.”
		“I guess I just breathe as best as I can and deep breathe now and realize that it’s going to be okay.”
		“The good doctor I had, he had all the pictures of his families and letters and stuff. It was so entertaining. Sometimes I hated getting seen too quick because it was like, I wasn’t done reading that.”
Communication	Communication-related challenges	It’s like I’m saying things to people in a language that’s misunderstood because it’s like a different language to them. I’m trapped in a no-signal zone. It’s like I’m a cell phone that’s working so properly but the signal is blocked, so that’s what it feels like.”
		“…I might not know until after I leave that, ‘Oh, I did not have this whole piece of information.’”
		“I mean, that’s often where I run into trouble is I’ll tell them I have pain, but I cannot explain is it stabbing or burning or that kind of thing because they all feel similar to me, so and then I cannot just tell them where a pain is, but then they will not give you an answer what’s wrong because it’s not a description of pain…They just do not look at it, so it comes off as it’s not a big deal. They just move on to the next thing.”
		“So if they send me a test result back and it says there’s a problem and I call to clarify what the problem is and they try to explain it, but they seem - like they seem to be raising their voice to me, I decide they are mad, and then I get upset with them that they are mad at me for asking questions and not - I’m not able to figure out the answer to my question.”
		“The most difficult part of primary care is “Trying to get an appointment to just meet [the doctor] face to face because there’s all these barriers of like, what number do you call? Or you get an 800 number that just feeds you into 1,000 directions, and talking on the phone is really hard for me. When I send [an online] message, they say, ‘Well, we cannot make the appointments. You have to call the appointment line’…Then you call the number…[and the automated phone line] gives you like three or four choices…And, honestly, you do not really know what [number] you are…if you think of probability and how many choices there are that you could possibly make, it’s like infinite…and then you get through, and then you are on hold for like 25 to 40 min waiting for a person to pick up…You talk to 10 people, and you give all your information 100 times. And then they try to fit you in, and it’s always 6 months past the date that you have even been sick. And you have to somehow verbalize something specific, and I do not know those words…And then they hang up, and you start all over again. So, it’s like this is insanity…There has to be one way to bypass for people who have disabilities who cannot do all that crap.”
		“He just does not seem to care…[it’s] hard to try when you feel like no one’s really listening, and it does not really matter anyway. He does not really worry about follow-up. He does not care if I never come back. He does not really care about getting to the root of the problem…it does not feel personal.”
	Desired provider communication styles	The doctor “Just came in and started a conversation… I’ve never had a conversation with a healthcare provider…I felt like wow. [Without this type of conversation, medical care is]…like a business, and the patients are on a conveyor belt that leaves less room for a human connection.”
		“There was this one doctor who was very calm and reassuring and buddy-like.”
		“Sometimes it was difficult to share what was wrong. And he would say, ‘It’s fine, take your time,’ and [he would] just sit down and…be really chill.”
		“I want a doctor who is assertive.”
		“I think if [your provider is] being upfront with you the whole time, it’s easier to be honest with yourself and honest to your doctor.”
		“I do not need the doctor to be warm and fuzzy. I do not need the doctor to be nice. I just need my doctor to give it to me straight. Yes, it’s an added perk if my doctor is empathetic and sympathetic and sensitive…But my number one priority would be just give it to me straight. No filter.”
		“I would need for them to break it down for me to make sure that I get it…simplify terms.”
	Strategies: identifying needed accommodations	“…I did not know how to advocate for myself because I never had accommodations. So, I was used to just sucking it up and failing. And [my doctor] actually came up with ideas originally…Once I had accommodations a little bit, I started to realize, there’s ways that I could do this that could be successful, and I should try to find out what they are, and he kind of helped me reach that.”
		“Like you might not be able to affect every doctor out there, but if you could empower every person and somehow make it available…like download these accommodations and questions, then you at least empower the person to do it themselves. Because while a doctor might not have the time for that in his mind, a client will make the time for that because we have to.”
	Suggested strategies	“My doctor knows that I think in pictures, so any time he’s trying to explain what’s wrong, he draws me a picture so that I can see the picture, and then he draws what’s happening on the picture for me.”
		My provider says “‘Every time you come in, you bring a list of complaints and things that has happened to you in the past 2 weeks. Everything that has gone on, I want all written down’…. And he tells me to just give it to him, and he goes through complaint to complaint with me, and also through concerns and positive things, and things that can change.”
		Post-visit printouts “…would be useful. Because a lot of times when I do take notes, like, I’m busy writing notes when I could be talking. You know, so that’s, like, missed time.”
		“He spends as long as I need to with him…Last time I went to see him, he spent over an hour with me. Even though he was already an hour behind, and it was 6:00 at night, he still stayed for an hour trying to figure out what was wrong…he did not care that it was going to make him go home late or that all the other medical staff had left.”
Autism-specific knowledge	Providers	After I disclosed my autism diagnosis to my provider “…it was a pretty poor attempt at hiding his facial expression. And I felt like saying – I said, ‘I’m not one of these people that just milks stuff.’ I mean, this [his symptoms] was legitimate, but I could tell I was not going to get through to him anyway. He made his formulation and opinion of me when I [disclosed my diagnosis]. And I basically wanted – I really wanted to say, ‘Doc, you are a shitty doctor,’ and then just walk out.”
		“There’s a commonality [among autistic people] to have certain types of dermatological problems, certain types of anxiety issues…GI issues, and the list goes on…[no] doc that I’ve talked to – including the current primary guy – is aware that my being on the spectrum, given my specific set of symptoms of things that I have going on, fits a pattern…So, I do not get that he’s going to be attuned to what other things might be going on that I’m not even aware of…if I were to tell the doctor, or the staff, that I’m on the spectrum, they do not get that I could have focus issues. They do not get that I could have balance or high eye-hand coordination issues.”
		“I think he has a close family member that is on the spectrum… it makes me feel good that they might know about me more.”
		“He did not really have much experience on my diagnoses …on my autism, or nothing - and he went out and educated himself, it looks like.”
	Office personnel	“She’s very kind of dismissive and brisk.”
		“The people were behind…the proverbial glass shutter instead of [being] open and feeling accessible.”
		“My doctor understands me very well, but I do not think the staff overall understand autism, especially, like, front desk…[or] the staff that does blood pressure…they just kind of take you back like they take any patient and then expect things to go smoothly…I always tell people what I need, but some of them go, ‘Oh, we do not need to do that,’ and then they wonder why you react negatively toward them.”
		“I just kind of flipped out…and the nurse just totally laid into me. She like completely had no clue what was my issue. Like she was just like, ‘Everybody has to wait. It’s too bad.’” She just like totally missed it…it was something like definitely related to autism that I melted down. And I tried to tell her, and she just came at me like some cranky woman like, ‘You need to calm down.’ Like I was being just a spoiled brat, like having a tantrum for waiting, and that wasn’t it at all. I did not mind being patient for [the doctor]. It was just the conditions were like really overwhelming, and I think it was really loud…But she just was really insensitive and mean, and that just sucked.”
	Strategies	“…watch the instructional videos. Read the books. Listen to the audiobooks, the instructions. Go to meet with people who either are the autistic people or those that work with them.”
		“[My PCP] spoke to my psychiatrist, he spoke to my neurologist…and he just started educating himself [about autism]…[Usually they] tell you ‘Sorry, I do not really handle this condition [autism].’”
Support	Support	“I often take my mom with me just so that I have a second person to hear everything that they say so that when I get home, she can repeat it to me and then I can translate it back into what I need because I cannot always process everything that’s said.”
		“I get a little overwhelmed by paperwork. But if I had somebody at the hospital or the office or the doctor break it down for me step-by-step, just to explain what everything is, then I’ll get used to it.”
		“Because I do not have a lot of support…I just have not gone…I used to see [my previous PCP] like every 3 or 4 months…[but] I’ve decided that I’m not going if I have to go alone…I need backup so that they do not bully me in an appointment.”
	Barriers to receiving support	The supportive living service provider industry “…has a huge turnover…I had one person, and then she quit. And then I had another person, and she walked off her job. And I had a third person, and she did not pass the background check. And now I have no one until they put someone else in place. So, I had someone for that appointment. Then they canceled that appointment. And then the person was pulled off the job, and now I need to wait for a new person [before attending my appointment].”
		The most challenging part of primary care is “Like it’s trying to get an appointment to just meet them face to face because there’s all these barriers of like, what number do you call? Or you get an 800 number that just feeds you into 1,000 directions, and talking on the phone is really hard for me. When I send a…message, they say, well, we cannot make the appointments. You have to call the appointment line… There’s no alternative…It forces you to have to get help from someone else, and then that - in some ways it like - it’s embarrassing. It’s like a humiliation. Like you are a capable adult. You just need this accommodated. But because they will not accommodate it, you then have to like ask for help, and it makes you feel like crap. You do not feel - you lose just a little bit of independence in that moment, and you are at the mercy of whether that person will call for you or not… And then they call for you, and then they say, we cannot talk to you. We can only talk to the person directly like because of HIPAA and all that stuff. And I’m like, I will waive all my HIPAA rights. Where do I sign? Like I do not even care about that. So that access, I think, just actually getting to the person that you are trying to interact with or to find out what to do from there or something.”

### 3.1 Finding a primary care provider

Multiple participants reported seeing the same provider for a significant length of time, ranging from 15 to 22 years, with one participant stating that “…once I found him, I stuck to him.” The number of years treated by the same provider contributed to patient satisfaction, based on the established relationship and trust that had developed, exemplifying the importance of long-term relationships in healthcare. This continuity of care was disrupted when participants had to find a new provider, most often due to the required transition from a pediatric to an adult PCP or the retirement of a current provider. Although many participants described how they “kind of got lost” when searching for a new PCP, they also noted the importance of considering objective and subjective characteristics when seeking to identify a new provider.

#### 3.1.1 Objective characteristics

Objective characteristics included insurance, office location, and transportation-related factors. Participants noted that insurance was essential, albeit “a hassle,” describing that “It’s not the doctors, it’s the insurance that has been [causing] a lot of trouble lately.” Ease of transportation access and location of the office were also critical in finding and choosing a doctor.

#### 3.1.2 Subjective characteristics

Subjectively, patients emphasized provider “reputation,” “experience,” and “good reviews,” often focusing on the desire to find someone who “understands me,” can “read me properly,” and who “I could build trust with.” However, it was often challenging to find a provider, with one participant explaining that “…we go through different doctors for different reasons. Either they did not understand me right or there was something not quite right…So it’s been a lifelong process I dare say all around.” In addition, participants emphasized the need to find a PCP who provided adequate care coordination, underscoring “how critically important it is to have a central medical person…to coordinate all the care,” explaining that “it’s really invaluable [having] one person kind of at the helm of what you are dealing with [to] kind of advocate for you.”

#### 3.1.3 Strategies

When actively engaging in the search process, participants described being “…just kind of on my own to find the new [PCP]. There was really no help…,” leading the majority of participants to seek recommendations or support from others. For example, many participants relied on family support, with one participant describing that “…my parents tend to help me try to find [doctors]…it’s still really hard to figure out the system.” Recommendations from family often led to using a family member’s provider, as one participant described, “I just went to my mom’s doctor because [my mom] was able to do it for me; then I did not have to try to figure it out because it’s too confusing.” Although still a challenging and often complicated process, participants also elicited and used recommendations from non-family members. For example, one participant described that

I talk to my typical developing friends…and if they did not like something that I know will trigger me, I avoid that one and then I keep asking. And then I’ll also begin to look online for reviews, and then once I find a set, I’ll ask my friends on the spectrum if they have heard anything about these doctors.

Utilizing the internet to identify a PCP was also challenging for participants due to the large quantity of information but dearth of details provided online, with one noting “there’s not really direct discussions of what you are looking for on websites or anything, so when you get there [the doctor’s office], it’s not what you expect.”

Once identified, participants described interviewing PCPs which “…did not always get me in the right place, but it eliminated a lot of bad apples.” One participant described how this process necessitated “…develop[ing] a format [of] how to question their office on the phone.” However, despite the perceived value of speaking with a provider prior to an initial visit, this strategy had its own challenges, including that “…I have not figured out how to interview a doctor without actually getting a medical appointment.”

### 3.2 Physical environment

Most participants reported that the physical space of the office, especially the waiting room, was “overwhelming,” “claustrophobic,” and “uncomfortable,” with the resulting discomfort causing one participant to “[not] get physicals for a while. I avoided the whole thing…Either the waiting rooms were chaotic, the wait times were chaotic, or I just wasn’t comfortable for any number of reasons.”

#### 3.2.1 Sensory sensitivities

The sensory components of the office and waiting room environments were reported to be noxious across most sensory modalities. Participants linked overcrowding to tactile sensitivities and increased stress, with one explaining that “I do not like being touched and I do not like having people super, super close to me.” Other distressing tactile experiences included the “crinkly paper” on examination tables and disposable patient gowns. Many participants also described problems with “the harshness” of visual stimuli, noting that fluorescent lights “give me a headache.” One participant described,

When I’d get in the room, it was really overwhelming, all the lights and everything. And so I’d always turn them off…[My new PCP] just turns them all on and does not even ask me if that was what I wanted…Like there’s no effort to make things easier or to make it doable.

The loud volume of the office was also problematic, with one participant noting that “you hear every little stinking thing.” Another described difficulty attending to verbal instructions when “you hear the rubber glove going [squeaking sound of rubber glove] and he’s making all these noises.” In addition, the “humming” emitted from fluorescent lighting and the noise from the paper on examination tables were reported to be distracting and bothersome.

##### 3.2.1.1 Strategies

Participants desired accommodations to navigate the sensory stimuli encountered during appointments. Informing medical professionals “in advance” about sensory preferences and/or discomfort with certain sensations allowed participants to request accommodations. While some felt reluctant communicating these suggestions to providers because “I know he will not change,” others were willing to “just tell them straight. It’s like, ‘Hey, this is what works for me, so just let me do this, and you do your job.’” Specific modifications suggested by participants included “dimmer” and non-overhead lighting, blue light to “help you relax and calm down a bit,” and/or wearing sunglasses. To mitigate auditory sensitivities, participants suggested headphones to drown out noises, or projecting white noise or “calming type nature music” in the office, but emphasized that “…it should not be very loud because loud would bother people on the spectrum…if it was too loud, it would overstimulate my sensory system.” To address tactile sensitivities, participants wanted PCPs to provide cloth gowns, permit participants to stay in their own clothing, and/or allow participants to sit in a chair instead of on the examination table. Additionally, a “sensory sensitive” quiet space in the primary care office that took “into account sensory triggers” was recommended; this did not have to be a separate space, but could be a “calming corner where it’s just quiet, kind of dimmer lights, and…separated off from the rest of the group.”

#### 3.2.2 Waiting

Challenges with the physical office space were exacerbated when participants had to endure lengthy wait times, described as “torture” by multiple participants. For example, one participant stated that “already I was [in the waiting room] for like 40 min, and then it was 45 min inside [the examination room]. And by then I was just like ready to lose my mind!”

##### 3.2.2.1 Strategies

When discussing their ideal primary care experience, almost every participant desired decreased wait times, with most considering 5–10 min to be reasonable. Participants often utilized scheduling-related strategies to minimize wait times and avoid crowded spaces. Participants also identified potential solutions to manage long wait periods. For instance, multiple participants suggested a notification system, “like when you go to those restaurants, and those things buzz when it’s your turn…[office staff could say] ‘You could go outside, and here’s this buzzer. Or even we’ll call you…do not worry. Go outside and I’ll call you.’ “The majority of participants valued “something just to distract you while you wait” to help “cope with anxiety and nervousness,” commonly utilizing technology to play games, watch videos and/or listen to music to support stress management and “kill time.” Participants wanted the office to provide items to support distraction, including reading material, TV, and/or “something like a fidget spinner…puzzle books…crosswords, Sudoku, that kind of stuff…Anything that passes time.”

### 3.3 Communication

#### 3.3.1 Communication-related challenges

Participants highlighted expressive communication difficulties during healthcare encounters, describing that “I’ve had trouble voicing my frustrations in a way that other people might understand” and “it’s really hard to find the right words to say and how to say them and when to say them.” Another participant described how “It’s like I’m saying things to people in a language that’s misunderstood because it’s like a different language to them.” Describing symptoms was especially problematic, highlighting the intersection of expressive communication and interoception difficulties. For example, one participant described that “I’ll tell them I have pain, but I cannot explain is it stabbing or burning…they all feel similar to me and then I cannot just tell them where a pain is.” Expressive communication challenges were compounded when “…you add melting down…I do not even know what comes out of my mouth half the time when I’m like that.”

Receptive communication also posed a barrier to care, with some participants describing that information provided by the doctor often “fl[ies] over my head” and “I do not always understand everything I’m told.” Another participant explained that

Sometimes someone will say something, and even though they’ll say it in that neurotypical way…that’s perfectly understandable to that person saying it and to other people they say it to, it might not click in for me…It may take two or three times to understand it, or it may be something that it may take months and then one day I’ll be thinking about it and then it’ll just suddenly click in right.

Often exacerbated by expressive and/or receptive communication challenges, multiple participants characterized their experience scheduling appointments as confusing, overwhelming, and frustrating, with one participant describing that the back-and-forth with automated systems and speaking with multiple staff was “insanity…There has to be one way to bypass [this process] for people who have disabilities who cannot do all that crap.”

#### 3.3.2 Desired provider communication styles

When describing qualities that were valued or desired in their PCP, participants predominantly focused on the importance of communication, with only a minority feeling comfortable asking questions and conveying their needs. Participants emphasized their need to feel listened to, with one explaining that, “…they listen to me when I say something. I mean they actually genuinely listen…I just want to be heard.” Participants wanted PCPs to communicate in respectful and nonjudgmental ways. For example, one participant noted “I’ve disclosed that I’m autistic…But they do not judge me any differently…they treat me with respect…I just ask to be treated like a human being and not dismissively.”

Participants valued their provider’s friendly and conversational approaches to communication, with one suggesting that this could prevent doctors from treating medical encounters “…like a business, and the patients are on a conveyor belt that leaves less room for a human connection.” Another explained that “I think a lot of it is just being more open and conversing…[before saying] ‘What’s bothering you today?’ “Similarly, participants appreciated gentle and patient communication styles, with one comparing his provider to Mr. Rogers and another recounting her provider’s response when she “melted down,” explaining that “I just felt like so like put at ease…he said, ‘Oh, do not cry. Let us try to figure this out.’ He was just so nice about it.” In contrast, another participant stated

I do not think [it’s too much to ask] for a doctor to just take a beat and be a little more patient when you are trying to choke out what’s wrong with you…Like they try to hurry, but then I never come back for my [next] appointment, so my healthcare suffers…It would’ve taken like 5 or 10 minutes extra just to be a decent person to have a successful appointment. Then I would not have to feel embarrassed, because I am.

Some participants valued “direct” communication, with one explaining that her PCP “does not sugarcoat” information. Another elaborated “I just need my doctor to give it to me straight. Yes, it’s an added perk if my doctor is empathetic…But my number one priority would be just give it to me straight. No filter.” In addition, most participants appreciated thorough and detailed explanations, with one participant valuing that “they really explain the details…he spelled out the steps…rather than me having me to figure [it] out.” However, a minority of participants reported that they “…bristle at too much detail” and would prefer providers to “simplify” communication.

#### 3.3.3 Strategies

##### 3.3.3.1 Identifying needed accommodations

To mitigate these mutual communication-related challenges, the majority of participants wanted providers to “automatically ask them, ‘What can I do to make your visit more comfortable?’” after the disclosure of their autism diagnosis. However, some participants reported they had not yet identified their own necessary accommodations, with one participant explaining that

…I did not know how to advocate for myself because I never had accommodations. So, I was used to just sucking it up and failing…Once I had accommodations a little bit, I started to realize, there’s ways that I could do this that could be successful, and I should try to find out what they are, and he kind of helped me reach that.

Because it was often “hard to know what you need,” multiple participants recommended that providers

…hand me a sheet [of possible accommodations to] help me be successful. And then I just check them off, and then they put that in my file, and they just work off that and then save me a bunch of torture and 3 years of trial and error. That would be great.

Although most participants desired their provider initiate these accommodations-related conversations, one participant recognized that their “doctor might not have the time for that in his head, but a client will make the time for that because we have to.” This participant emphasized the need to “empower every [autistic] person” to utilize publicly available lists of potential accommodations as a method to inform providers of their individualized needs prior to care. These types of tailored strategies were noted as especially important because “…each person has different needs and [providers need to] learn to explore that.” Provision of non-tailored strategies were reported to be problematic, with one participant describing that “cookie cutter accommodations do not help…[they] can just almost make things worse.”

##### 3.3.3.2 Suggested strategies

Participants emphasized that providers should “answer things in that alternative way” or “phrase things a little differently” to enhance patient understanding. Providing visuals and/or written instructions also aided communication. For example, “My doctor knows that I think in pictures, so any time he’s trying to explain what’s wrong, he draws me a picture.” Participants also explained the benefit of written summaries because providers “in 30 s – tell you everything you need to do…For years I just would just smile and go, okay, all right, great, and then walk outside and say to the nurse, ‘What does he want me to do?’” When PCPs provided printed or written information “It was just so much easier. It was like he just kind of filled the gap, so that I could go and be independent and successfully have an appointment.”

Multiple participants also described the importance of providers preparing the patient for what was to come in the healthcare encounter. Without these “warnings,” participants felt “blindsided” which “results in more anxiety.” When providers “forget to tell me they are going to do something…[I] get upset, and then I miss half of what’s said.” These strategies often mitigated tactile-related sensory sensitivities, with participants appreciating when providers “warn me before they touch me…So then it does not feel like it’s sending my tactile system through the roof.”

Lastly, to alleviate scheduling-related challenges, participants appreciated when providers offered alternatives to communicating via telephone calls, including one-on-one assistance at the end of appointments, scheduling via text message, or utilizing online scheduling systems.

### 3.4 Autism-specific knowledge

#### 3.4.1 Providers

Providers’ lack of autism-specific knowledge had the potential to contribute to problematic health care encounters. For example, following disclosure of his autism diagnosis, one participant recounted that his PCP had

…a pretty poor attempt at hiding his facial expression…[my symptoms were] legitimate, but I could tell I was not going to get through to him anyway. He made his formulation and opinion of me when I [disclosed my diagnosis]…I really wanted to say, “Doc, you are a shitty doctor,” and then just walk out.

Participants explained that providers’ understanding of how to best treat autistic patients is limited by the societal focus on autism which “is mostly about children. You know, let us face it. It really is. It’s now only maybe now a little bit about adults.” Participants wanted providers to have “a lot more understanding of the, of the people who are on the spectrum and their habits that they might have. Which they are not always bad…There’s some that are just different.” Without sufficient knowledge about autism, PCPs “…mostly do not know what they need to accommodate…[it’s] mostly out of ignorance.”

#### 3.4.2 Office personnel

Non-physician personnel were frequently described as “insensitive” and “dismissive.” Multiple participants described feeling frustrated when they advocated for needed modifications and were met with “‘Oh, we do not need to do that’, and then they wonder why you react negatively toward them.” Another participant described that “I could not stop pacing…So [office staff] asked me to leave…[I said] ‘I need to leave the waiting room, because you do not allow me to pace here. Then I’ll be in the hallway’…[but they said] they were not going to call me outside,” negatively impacting his access to care. Other participants stated that after a positive encounter with their PCP, the office staff often “destroys it again when I leave…because people do not know how to communicate that aren’t trained.” This was further exemplified when one participant described an interaction with a nurse following a long wait,

…I just kind of flipped out…And the nurse just totally laid into me. She like completely had no clue what was my issue…it was something like definitely related to autism that I melted down. And I tried to tell her, and she just came at me like some cranky woman like, “You need to calm down.” Like I was being just a spoiled brat…It was just the conditions were like really overwhelming, and I think it was really loud…But she just was really insensitive and mean, and that just sucked.

#### 3.4.3 Strategies

To address these challenges and problematic encounters, participants suggested that “…some sort of education to train *everybody* on how to deal with those on the spectrum would be good.” Participants asserted that education could prevent discriminatory behavior, support understanding of individualized needs, and improve healthcare encounters. While education was highlighted as essential, participants also underscored the importance of understanding that “no two people with [autism] are the same…even if you have all the training in the world, you are not going to know what one person from the next needs.” Another participant echoed this sentiment, explaining that “I do not think teaching [PCPs] how to *do* certain things is the answer. I think it’s teaching them how to *ask* what will be helpful” [emphasis added]. Patience, diligence, and trial and error were often required to “figure out over time what [was] getting in the way” in order to identify and then implement changes to improve healthcare encounters.

### 3.5 Support

Incorporating a support person – such as a family member, friend, and/or “supportive living service provider” – into primary care was most often utilized to support challenges with communication. Multiple participants described being “not very good at relaying verbal information” and requiring assistance to avoid giving their PCP “an incomplete picture of the story.” One participant elaborated that her support person is “better at explaining herself…Whereas I tend to clam up and think about it in my head, but I do not speak about it.” Another described that it was valuable to “have a second person to hear everything that they say so that when I get home, she can repeat it to me…because I cannot always process everything that’s said.” Other participants described benefiting from help filling out “overwhelming” paperwork or scheduling appointments, explaining that “I usually have someone else [schedule appointments] or I do not do it at all.” In addition, some reported feeling uncomfortable, anxious, and unable to advocate for themselves without a support person, with one participant describing that “I’ve decided that I’m not going if I have to go alone…I need backup so that they do not bully me in an appointment.”

#### 3.5.1 Barriers to receiving support

However, incorporating a support person was not without barriers. For example, taking into consideration three busy schedules instead of two provided logistical challenges, especially when a PCP or support person (either family member or a hired support person) had to reschedule. For example, one participant described that after her PCP canceled her appointment, “I had to cancel childcare. I had to cancel the person coming with me. I had to – it just kind of like affects a lot of stuff.” Another participant reported that because the supportive living service provider industry “has a huge turnover,”

…I had one person, and then she quit. And then I had another person, and she walked off her job. And I had a third person, and she did not pass the background check…now I need to wait for a new person [before attending my appointment].

Once scheduling barriers were overcome, the most commonly reported challenge was when PCPs directed their attention to the support person instead of the autistic patient, with one participant describing that “…the doctor talked to her and not to me. And if I wasn’t so beaten down and depressed at the time, I would’ve probably said, ‘Excuse me, doctor. I’m over here.’…It was just incredibly disrespectful.” Others described privacy-related difficulties, due to either office regulations or their own desire for keeping personal health information confidential. In the office, one participant recounted how his doctor “was saying that adults could not have their parents with them.” Another described that scheduling difficulty “forces you to have to get help …It’s like a humiliation…And then they finally call for you, and then say ‘We cannot talk to [the person helping] you. We can only talk to [you] directly, like because of HIPAA and all that stuff.’” Participants also reported that incorporating a support person compromised their desired privacy, with one explaining, “I do not want to tell my whole life or whatever private thing is going on for me in front of a practical stranger that, yes, is helping me, but I do not even know who they are…I would not even want my mother to come with me…Like, you want to be able to share what’s happening with privacy.” Despite the value of a support person, it was “…a mixed bag. I’m glad that somebody’s there, but it’s also embarrassing to have somebody there.” Furthermore, participants expressed frustration that the healthcare encounter was not tailored for independent success, explaining that

I would rather have a private meeting with my doctor and have them be capable of helping me in a way that does not make it worse. And I do not think it’s asking too much…I would like to be able to do that myself and not have to be dependent on someone else coming with me.

## 4 Discussion

Findings suggest that autistic adults face multiple barriers to receiving and accessing primary care, with the most prominent difficulties relating to the physical environment and communication.

### 4.1 Physical environment

It is well understood that autistic people experience sensory processing difficulties across the lifespan (33, 34). Unfortunately, the primary care environment is often laden with sensory-based triggers across all modalities. Sensory stimuli have been reported to be problematic for autistic patients starting in the waiting room (23, 35) and persisting throughout the medical encounter, with our participants and those in previous studies emphasizing challenges with tactile, visual, and auditory stimuli (24, 26, 36–38), as well as olfactory stimuli (24, 36, 37) which was not described by our participants. These sensory challenges have been reported to have serious downstream implications, and are associated with untreated health conditions and the need for additional medical treatment (23).

Reducing stimuli may decrease stress and improve information processing for autistic adults (38). Many of our participants’ suggestions support this and align with those previously reported, including allowing patients to wait in a quiet area or outside and minimize overstimulating wait periods (13, 23, 24, 36, 38), use a white noise machine to dampen loud noises (24), and/or replace fluorescent lighting (13, 24, 38). Successful distraction techniques, emphasized by our participants and others, may include the use of sensory tools such as fidgets (23).

### 4.2 Communication

Communication-related challenges were also overwhelmingly endorsed by our participants, with the discordance between communication styles of autistic patients and non-autistic PCPs often at the forefront of problematic healthcare encounters. These findings are consistent with the double empathy theory, which frames communication challenges as mutually and interpersonally situated between autistic people and often non-autistic healthcare providers (39). Previous research has similarly described mutual communication challenges during healthcare encounters for autistic adults, specifically regarding difficulties making appointments (23, 36, 37), feeling understood by providers (23, 35, 40), answering open-ended questions (13, 36–38), and having adequate time to process information (36, 38). Similarly, 73% of surveyed physicians reported challenges communicating with their autistic patients (41). Communication difficulties are associated with multiple adverse health outcomes for autistic adults, including missing appointments, untreated health conditions, and requiring additional medical treatment (23).

Strategies implemented by providers to improve communication with autistic patients include using direct language (23), avoiding open-ended questions (13, 36), supporting written communication (24, 36, 38), providing time for patients to ask questions (36), warning patients about what is to come (36), and actively confirming patient understanding (36, 38). Despite supporting the majority of the above strategies, participants in this study expressed many opposing communication preferences (e.g., desiring thorough vs. concise communication). Due to this heterogeneity, our participants suggested tailored approaches to meet individualized needs and improve communication; this could be accomplished by utilizing interventions such as the Autism Healthcare Accommodations Tool, a web-based resource for autistic patients to generate a customized list of accommodations that can be shared with providers (42).

### 4.3 Sensory and communication

As described by our participants and supported by previous research, there exists a multi-directional interplay between sensory and communication-related healthcare barriers. For example, communication challenges can be exacerbated by sensory discomfort, impacting attention, language processing, language production, and social interaction, thereby potentially hindering a successful healthcare encounter (23, 25, 38, 40, 43). In addition, problems communicating symptoms to providers may be compounded by difficulties with interoception, one’s awareness of internal bodily sensations and cues such as pain (44). Although interoceptive challenges have been linked to eating disorders, alexithymia (i.e., difficulties with recognizing and expressing emotions), and anxiety (45), little attention has been paid to implications for primary care encounters.

### 4.4 Autism-specific knowledge and stigma

Beyond the highly endorsed challenges with the physical environment and communication, this study also reinforces the reports of autistic adults regarding providers’ lack of sufficient autism-specific knowledge (13, 24, 35, 37, 46), a perception that is corroborated by providers themselves (20, 21, 26, 46, 47). Autistic adults have linked this lack of knowledge to misconceptions and discrimination during healthcare encounters (14, 35, 37), potentially negatively impacting provision of care (46). For example, some autistic patients choose not to disclose their diagnosis to providers because of these concerns (37). In one study, almost half of autistic adults who disclosed their diagnosis were then questioned by providers regarding that diagnostic accuracy (38). These findings and problematic encounters highlight providers’ insufficient understanding of the heterogeneity in clinical presentations of autism. This is consistent with the neurodiversity model of autism, which frames autism as a marginalized minority identity and calls for addressing the discrimination and disparities autistic people widely experience (48).

Both autistic adults and PCPs have reported desiring autism-related training for providers (13, 20). One intervention to target this knowledge gap is the Extension for Community Healthcare Outcomes (ECHO Autism), in which physicians are educated by autism experts on the diagnosis and treatment of autistic patients (16, 49). However, many autistic adults have reported that as long as providers have a rudimentary understanding of autism, it is more important to understand how heterogeneous autism can be (38). Therefore, recognizing and respecting this diversity of needs and preferences is essential in providing effective support, in addition to further education.

### 4.5 Support

Many autistic adult patients desire involvement of a support person during healthcare encounters (e.g., 13, 37). In addition to the reports from our participants, previous research explicates that effectively incorporating supporters into care enhances accessibility, communication, visit success, and patient satisfaction (13, 18, 35). However, incorporating a support person is not without significant barriers. For example, our participants and autistic adults in previous research often reported that providers over-prioritized supporters in healthcare encounters, at the expense of attending to the patient (13, 18, 50).

Support people have also expressed barriers to their involvement in healthcare encounters, expressing frustration when prohibited from attending appointments or communicating with providers (18, 51). When permitted to attend appointments, support people often felt ignored and dismissed when advocating for the healthcare needs of autistic adult patients (18, 52). In addition, PCPs acknowledge the difficulty of managing the involvement of a support person, describing that having “two patients” instead of one is not something they are accustomed to (20).

### 4.6 Limitations and future research

While research paints an increasingly vivid picture of the barriers and facilitators that autistic patients face when receiving care, it is important to note that these results represent only the experiences of the recruited participants. This study collected only limited demographic and descriptive information from participants (e.g., did not collect information related to IQ, autism severity [i.e., DSM-5 three diagnostic levels of autism severity/support needs (2)], insurance/housing/employment status), which limits the contextualization of findings for potentially different cohorts as these factors have the potential to influence the primary care health experiences for autistic adults. In our efforts to obtain greater depth and breadth of information about primary care experiences, participants were not instructed to answer questions about only their current care; instead, they were allowed to reflect on both their current and previous primary care experiences in their answers.

Multiple gaps in this literature warrant further research. For example, the intersection of race/ethnicity and autism in healthcare for autistic patients has been insufficiently explored (53). Despite efforts to recruit a diverse sample, a limitation of this study is that only 22% of participants self-identified as an under-served race/ethnicity; these participants did not share any experiences about race/ethnicity impacting care, in direct contrast to our sample of caregivers of autistic adults (18).

Additional research is also warranted to support the development and testing of interventions to improve primary care for autistic adults. As the population of autistic adults continues to grow (27), the importance of prioritizing first-hand autistic perspectives throughout all phases of intervention development and evaluation cannot be overstated. Despite the inclusion of two autistic co-researchers in the development and execution of this study, neither chose to participate throughout the analytic phase, a limitation of our study, although another autistic co-researcher assisted with the study framing, language use, and manuscript writing. Gathering information from support people and providers may also contribute insight into the development of interventions. Interestingly, there are striking similarities between these three informant groups when describing barriers to primary care for autistic adults, shining a spotlight onto potential foci for intervention, including: patient-provider communication, the physical environment, providers’ autism-specific knowledge, and incorporating supporters in care (e.g., 13, 18, 20, 47), all while emphasizing the importance of individualization of care to maximize successful healthcare encounters (e.g., 13, 18, 20).

Challenges for autistic adults, such as those reported by our participants, are not limited to the primary care environment, but also pervade experiences in other settings, including hospitals (54), emergency departments (55), dental offices (56), and the workplace (57). Interventions to mitigate barriers in these other settings support recommendations from this study. Future research should harness information across these disparate settings to develop interventions that may be employed in multiple environments.

## 5 Conclusion

As described by autistic adults, there are significant barriers to accessing and receiving primary care, most notably the physical environment and communication. Participants suggested strategies to mitigate these challenges, many of which are practical, low/no cost, and easy to implement. Strategies also emphasized the diversity of experiences and preferences for autistic patients, highlighting the importance of tailoring accommodations in the primary care setting.

## Data Availability

The dataset presented in this article are not readily available because our study analyzed qualitative data which contain potentially identifiable information and participants did not consent to having their full transcripts made publicly available. Due to ethical restrictions imposed by our IRB surrounding sensitive data and participant privacy concerns, we are unable to make the full transcripts available publicly. Excerpts of the transcripts relevant to the study are available to anyone who contacts us with this request. Requests to access the dataset should be directed to lstein@chan.usc.edu.

## Footnotes

^1^We would like to acknowledge that there are various and evolving perspectives regarding the use of identity-first (i.e., autistic adult) versus person-first (i.e., adult with autism) language. While there is no consensus on this highly personal choice (58), research suggests that the majority of autistic adults, parents of autistic individuals, and autistic advocates prefer identity-first language (e.g., 59, 60), suggesting that identity-first language “de-pathologize[s]” autism (61, p.1) and does not separate autism from the individual, instead positioning autism as inextricably linked to one’s core identity (59, 62). For these reasons, the authors of this paper have decided to respect this expressed preference and use identity-first language.

^2^In this definition, we refer to autism diagnostic criteria outlined in the Diagnostic and Statistical Manual of Mental Disorders-5 (2). However, in this manuscript we employ neutral terminology (e.g., autistic “characteristics” vs. “symptoms”), consistent with autistic community language preferences and emergent autism research best practice recommendations (29, 48).
