# Supplementary material for: Barriers and facilitators to primary healthcare encounters as reported by autistic adults: a qualitative study
Source: Front Med (Lausanne). 2025 Mar 12;12:1481953. doi: 10.3389/fmed.2025.1481953 (PMC11937071; doi:10.3389/fmed.2025.1481953)
Supplement: Supplementary file 2 [file Table_2.pdf]

1.2 **Supplementary Table 2.** Descriptive characteristics of participants, stratified by recruitment location.

	Autistic Participants: Los Angeles (n=17)	Autistic Participants: Philadelphia (n=15)
	**Mean (SD) / Range**	**Mean (SD) / Range**
**Age**	30.6 (11.60) / 18-57 years	33.2 (13.1) / (21-67 years)
**RAADS-R Score**[a]	116.1 (55.5) / 65-224	124.8 (44.0) / 66-200
**All sources considered, how much money do you have access to monthly?**[b]	$1,596.92 (1,572.04) / $0-$5,000	$1,408.50 (754.68) / $200-$3,000
	**N (%)**	**N (%)**
**Sex**		
Male	14 (82.4)	12 (80.0)
Female	3 (17.6)	3 (20.0)
**Race**[c]		
White, Caucasian	11 (64.7)	12 (80.0)
Black or African American	2 (11.8)	2 (13.3)
Asian	2 (11.8)	2 (13.3)
American Indian or Alaska Native	3 (17.6)	0 (0.0)
Not Reported	1 (5.9)	0 (0.0)
**Ethnicity**		
Not Hispanic, not Latino	12 (70.6)	14 (93.3)
Hispanic, Latino	5 (29.4)	1 (6.7)
**Primary Language Spoken in the Home**		
English	14 (82.4)	14 (93.3)
Spanish	2 (11.8)	0 (0.0)
More than one primary language[d]	1 (5.9)	1 (6.7)
**Highest Level of Education Earned**		
High School or GED	6 (35.3)	5 (33.3)
College	9 (52.9)	9 (60.0)
Graduate Degree or above	2 (11.8)	1 (6.7)

Note. RAADS-R = Ritvo Autism Asperger diagnostic Scale-Revised.

[a]Autism diagnosis confirmed either by medical documentation or RAADS-R; n=15 RAADS-R scores included for Los Angeles participants and n=8 RAADS-R scores included for Philadelphia participants.

[b]Missing data (n=4 Los Angeles; n=5 Philadelphia)

[c]Participants instructed to mark all that apply.

[d]Los Angeles: English and Cantonese (n=1); Philadelphia: English and Spanish (n=1)
